# A Systematic Literature Review of Three Stenting Strategies for Bifurcation Lesions in Coronary Artery Disease

**DOI:** 10.36469/9746

**Published:** 2019-04-26

**Authors:** Larragem Parsley-Raines, Dominique M. Brandt, Dillon L. Carr, Sabrina Uhry, Eileen S. Alexander, Stephanie A. Donauer, Peter J. Mallow

**Affiliations:** 1 Xavier University, Cincinnati, OH; 2 Centre Hospitalier de Haguenau, Haguenau, France

**Keywords:** provisional strategy, Tryton^®^ Side Branch Stent, bifurcation lesion, complex strategy, target vessel failure (TVF), major adverse cardiac event (MACE)

## Abstract

**Background:**

Bifurcation lesions represent 15–20% of all patients undergoing a percutaneous coronary intervention (PCI) for coronary artery disease. The provisional 1-stent stenting strategy is the preferred strategy to treat bifurcation lesions. Other strategies used to treat bifurcation lesions include 2-stent complex stenting strategies and the Tryton Side Branch Stent^®^ (TSB)—a dedicated side-branch stent for bifurcation lesions, which gained FDA approval in March 2017.

**Objectives:**

To conduct a systematic literature review of the safety and effectiveness of three stenting strategies (provisional, complex, and Tryton Side Branch Stent^®^) for bifurcation lesions with a side-branch diameter ≥2.25 mm, undergoing PCI.

**Methods:**

Literature searches in Medline, Cochrane Library, Web of Science and Embase were conducted to identify prospective clinical trials from January 2007–July 2017.

**Results:**

602 articles were identified. Nine articles (6275 patients) met all inclusion criteria. Seven studies (5282 patients) compared provisional to complex stenting strategies. Two studies (993 patients) compared provisional to the TSB. Outcomes of interest reported were target vessel failure in 2 studies, major adverse cardiac event (MACE) (cardiac death, all myocardial infarction, ischemic driven target legion revascularization TLR) in 5 studies. For target vessel failure, the provisional strategy ranged from 5.6% to 15.6 %; complex at 7.2% (one study); and TSB from 11.3% to 17.4%. For MACE, provisional strategy ranged from 8%–13.2%; complex from 11.9%–15.2%; and TSB from 8.2%–18.6%.

**Conclusions:**

To our knowledge, this is the first review comparing three bifurcation lesion stenting strategies. Significant heterogeneity in the study design of the nine studies reviewed prevented a meta-analysis. A clinical trial comparing the TSB to both the provisional and complex strategies would provide better inference on the safety and effectiveness when comparing strategies.

## Background

Approximately 20% of coronary artery disease (CAD) patients who undergo a percutaneous coronary intervention (PCI) have a bifurcation lesion, e.g., a plaque buildup at the crux of the main branch (MB) vessel and its side branch (SB) vessel.^1^ Among all types of lesion subsets, bifurcation lesions are considered one of the most challenging and difficult to treat due to complex anatomical factors and corresponding high rates of adverse events.^2^,^3^ Different strategies for stent placement are performed specifically for bifurcation lesions, including the provisional, 1-stent approach, and the complex, 2-stent approach.^4^ The provisional stenting strategy is currently considered the recommended strategy for treating bifurcation lesions.^5^ The provisional stenting strategy uses a single drug-eluting (DES) stent or bare-metal (BMS) that is deployed in the MB vessel. Alternatively, the complex stenting strategy utilizes two stents (either DES or BMS), to alleviate blockage in both the MB and the SB vessel.^4^,^6^ A 2014 meta-analysis compared provisional and complex stenting strategies using DES stents and found complex strategies to be superior to provisional strategies for bifurcation lesions when the diameter of the SB was ≥ 2.5 mm. However, the data did not support overturning the consensus that the provisional strategy is the recommended approach.^7^ The anatomy and the severity of the lesion, are important factors to take into account when deciding whether to use a provisional or a complex strategy.^8^ A 2015 clinical trial found that patients undergoing PCI with true bifurcation lesions—defined as lesions affecting both the MV vessel and the ostium of the SB, Medina classification 1, 1, 1; 1, 0, 1; or 0, 1, 1, also involving a SB reference vessel diameter (RVD) of ≥ 2.3 mm—had worse clinical outcomes than patients without true bifurcation lesions.^2^,^9^ The authors strongly recommend differentiating the two types of bifurcation lesions in future studies.^9^

The Tryton Side Branch Stent^®^ (TSB) received FDA approval in March 2018, and it is the only dedicated SB stenting strategy for a bifurcation lesion with a SB diameter of ≥ 2.25 mm.^10^ This approach deploys a BMS in the SB vessel and subsequently a DES in the MB vessel allowing for coverage in the SB, MB, and transition zones.^11^ The TRYTON Bifurcation Study, a multicenter controlled clinical trial of 704 patients, compared the TSB to the provisional stenting strategy. This study demonstrated an 18% reduction in SB in-segment diameter stenosis among patients treated with the TSB compared to patients treated by the provisional stenting strategy.^12^

The objective of this study was to evaluate the safety and effectiveness of the provisional, complex and TSB stenting strategies for bifurcation lesions in PCI caused by CAD. This systematic literature review adds to the literature by including TSB as a third stenting strategy.

## Methods

### Literature Search

A literature search was conducted using Medline, Cochrane Library, Web of Science and Embase to identify all relevant articles from January 2007 to June 2017. Search terms used for each database can be found in Appendix A.

### Inclusion and Exclusion Criteria

The inclusion criteria were as follows: (1) Study was published in the English language; (2) Study was prospective; (3) Study included a comparison of provisional to complex strategies or provisional to TSB; (4) Duration of patient follow-up was ≥ to 6 months; (5) bifurcation lesion was defined as SB with a RVD≥ 2.25 mm as determined by either quantitative coronary angiography or visual assessment; (6) Study included human subjects; (7) Study was published between January of 2007 and July 2017. The exclusion criteria were as follows: (1) Study was retrospective; (2) No patient follow-up, or follow-up was < 6 months.

### Data Extraction and Quality Assessment

Data extraction was conducted by the investigators and involved capturing various data elements from each paper identified by the search terms (Appendix B). Two reviewers extracted the data based on the inclusion and exclusion criteria. From those results, the quality of the study was assessed as either: strong (zero weak ratings), moderate (one weak rating), or weak (two or more weak ratings). Ratings were determined using questions from the Quality Assessment Tool for Quantitative Studies.^13^ Once global ratings were established, they were discussed by both investigators. In the event of a rating discrepancy, the disagreement was documented, and a third investigator served as referee.

### Study Endpoints

The primary endpoints included Target Vessel Failure (TVF) - a composite index of cardiac death, myocardial infarction (MI), and clinically indicated target lesion revascularization (TLR) and target vessel revascularization (TVR) - and Major Adverse Cardiac Event (MACE) - clinically indicated cardiac death, MI, TVR and TLR (Table 1). Other endpoints that were extracted were TVR, and stent thrombosis (ST): both definite + probable.

## Results

### Literature Search

**Figure 1:**
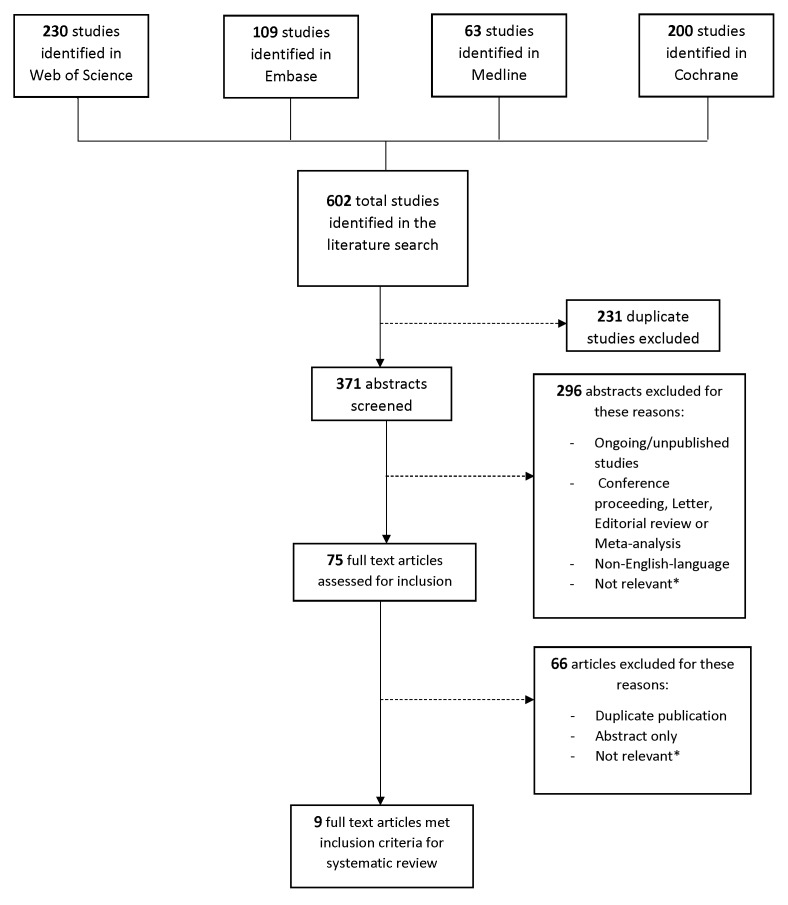
Attrition Diagram of Systematic Review

The literature search identified 602 studies. Among these 602 studies, 231 were duplicates, leaving 371 studies to be screened. Among the 371 remaining studies, 296 were excluded. These excluded abstracts included ongoing studies, conference proceedings, letters, editorial reviews or meta-analyses, non-English language abstracts, or abstracts that were considered not relevant to the subject. Following this process of abstract review (defined as Level 1 screening), 75 studies were eligible for full article review (Level 2 screening). Of the 75 articles, 66 were excluded for not meeting the defined inclusion criteria. The nine remaining studies (a total of 6275 patients) met all inclusion and exclusion criteria. Seven studies (5282 patients) compared provisional to complex stenting strategies. Two studies (993 patients) compared provisional to TSB stenting strategies. Based on the quality assessment of all nine studies, according to the Quality Assessment Tool for Quantitative Studies, 8 studies were considered to be of moderate quality, and one study was considered to be of weak quality^13^ (Figure 1 and Table 1).

### Patient Characteristics

Patients enrolled in the 9 studies shared similar demographic characteristics being predominantly male (73.4%–85%) with a mean age ranging from 62.3 to 68.0 years as seen in Table 1. The comorbidity profiles of patients in the 9 studies varied. All studies reported the proportion of subjects with hypertension, previous MI and diabetes; all but Chen *et al*., 2017 reported on current smokers.^14^ In Ferenc *et al*., 2008, there was a higher percentage of patients with hypertension (in both the provisional and complex arm 92.1% and 89.1 respectively).^15^ Conversely, in Hildick-Smith *et al*., 2010, the percentage of patients with hypertension was lower (in both the provisional and complex arm 57% and 62% respectively).^16^ Finally, the study by Hildick-Smith *et al*., 2016, had a higher than average proportion of smokers.^17^

### Stents Used

All nine studies were conducted using DES: Three studies used a first-generation DES (Stainless steel platform)^15^,^16^,^18^; three used a second-generation stent (biodegradable polymer platform)^14^,^17^,^19^; one used either a first, second-generation (cobalt chromium platform) or (biodegradable polymer platform) stent^20^; and two used the TSB stent (cobalt chromium) with DES.^12^,^21^ DES were Sirolimus in four studies^14^,^15^,^18^,^19^; Paclitaxel in one study^16^; Biolimus in one study^17^; Sirolimus, Paclitaxel, Everolimus and Zotarolimus in one study^20^; two studies did not specify which drugs were used.^12^,^21^ See Table 2.

### Reported Outcomes

Outcomes were only reported when a comparison was possible among the three strategies. Chen *et al*., 2017 was the only study that reported an outcome with 5-year follow-up and was not used in our comparative analysis.^14^ Primary outcomes reported were TVF in two studies.^12^,^21^ MACE in 5 studies,^14^,^15^,^18^–^20^ and two different composite indexes at 9 and 12 months with TVF and TVR, respectively.^16^,^17^ Results for the two composite indexes were not used in our comparative results. See Table 1.

**Figure 2:**
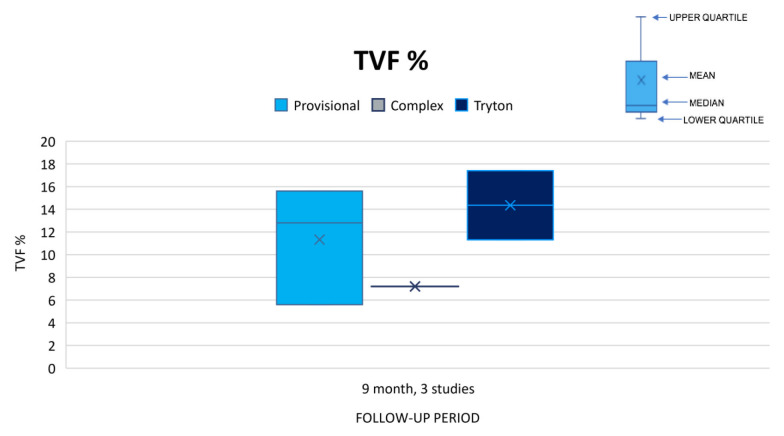
Studies reporting TVF (%) at 9 months. TVF: target vessel failure

**Figure 3:**
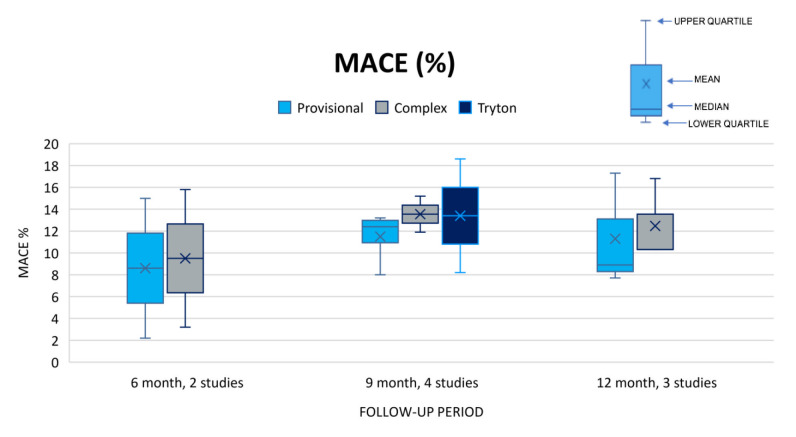
Studies reporting MACE (%) at 6, 9 and 12 months. MACE: major adverse cardiac event

When combining all primary and secondary endpoints we obtained the following results: Three studies reported TVF at 9 months with the provisional strategy ranging from 5.6% to 15.6 %, TSB from 11.3% to 17.4%, and complex at 7.2%.^12^,^16^,^21^ The lowest results in the provisional and complex strategies were found in the Hildick-Smith *et al*., 2010 study.^16^ No strategy was found to be superior for TVF at 9 months. See Figure 2. MACE was reported at 6 months, 9 months and 12 months for five of the included studies, however comparison of the 3 stenting strategies was only possible at 9 months for 4 studies.^14^,^15^,^18^–^20^ For MACE at 9 months, results for the provisional strategy ranged from 8.0%–13.2%, TSB from 8.2%–18.6%, and the complex strategy from 11.9%–15.2% respectively (Figure 3).

## Discussion

To our knowledge, this is the first systematic literature review to compare three stenting strategies—provisional, complex and TSB. Previous systematic reviews and meta-analyses have compared the provisional to complex strategies, but did not provide clear recommendations as to how the intervention should be implemented.^7^,^22^–^24^ Studies conducted by Nairooz *et al*., 2017, Zhang *et al*., 2009, and Hakeem *et al*., 2009, concluded that the provisional strategy was more likely to be associated with improved short and long-term outcomes.^22^–^24^ After conducting a subset analysis, Gao *et al*., 2014 recommended the complex strategy as an optimal treatment for a bifurcation lesion with a large SB vessel of at least 2.5 mm in SB diameter.^7^ Most meta-analyses published had reservations about the various types of complex techniques such as Culotte, Double Kissing (DK) Crush. Various complex techniques may have a different impact on measured outcomes.^22^,^24^,^25^ Current stents used to treat bifurcated lesions have been designed to cover straight vessel lesions and interventional cardiologists need to innovate in order to cover bifurcated lesions. Complex strategies are more dependent on the skills and experience of the interventionist (Table 2). This type of stenting procedure is longer and often requires stent distortion to fit the lesion.^26^ Dedicated stents, such as TSB, are designed to control for difficulties related to standard PCI, notably to perform the procedure without having to rewire the SB.^26^, ^27^

So far, only two studies have been published on the safety and efficacy of TSB. After conducting the first TSB trial in 2015, Généreux *et al*. discovered that they had inadvertently enrolled patients with a smaller SB diameter caused by inconsistencies amongst interventional cardiologists when choosing their method of measuring the SB, e.g. Visual assessment vs. QCA(12). The authors conducted a second study in 2016 with careful measurements of SB diameters. Results showed a reduction in MACE from 18.6% in the 2015 study to 8.2% in the 2016 study for the TSB comparator. Similarly, a steep drop was observed in the TSB branch for MI (15.1% and 9.2%), respectively.^21^

Our study was designed to compare three stenting approaches for bifurcation lesions and to shed some light on the controversy regarding the appropriate strategy. Among the large pool of studies selected in our search, only nine studies fit our criteria for inclusion into a comparison among the three strategies. A limiting factor was the specific inclusion criteria of the SB being ≥ 2.25 mm. Studies had various follow-up periods and comparison among all strategies was only possible for a 9-month follow-up period. For the comparison of safety, primary outcomes MACE and TVF, TSB did not appear to perform better than the two other strategies. However, no cardiac death was observed in the TSB group compared to the two other techniques, but patients treated with TSB had higher risk of MI and stent thrombosis than if treated otherwise.

When comparing efficacy among stenting strategies (TVF and TVR), results were highly variable for provisional versus the two other strategies. TVF and TVR were also lower in the TSB for the 2016 study by Généreux *et al*., compared to the 2015 study (3.5%–4.9% and 3.5%–5.5%) respectively. As the controversy persists it is important to keep in mind that the anatomy and severity of the lesion is an important factor when choosing a stenting strategy. Comparative studies should include anatomic variations, bifurcation angle and severity of the lesion in the SB. In the absence of patient level data, comparative studies provide only limited information on which strategy to choose.

Studies used in this systematic review had substantially different patient populations, devices used, and study design, therefore progression to meta-analysis was not possible.^28^ Patients enrolled in the nine studies used for our analysis shared concerning similarities in age and gender proportions. However, comorbidities did vary substantially amongst our patient population. A meta-analysis assumes that studies are similar in population characteristics such that individual studies are like samples drawn from the same population. This was clearly not the case in the nine studies identified in our systematic review.

Second, differences were observed among study designs. The nine studies analyzed used different primary endpoints to determine study sample size. MACE was the preferred primary outcome. TVF was only used in studies comparing TSB to provisional and a different composite index was used for the two Hildick-Smith *et al*. studies. In addition, there was no clear consensus in terms of follow-up period and not all studies reported the specifics on medication prescribed after the intervention. It also important to note that during the period in which the studies were conducted, stenting technology was and continues to rapidly evolve with a multitude of new DES’ to choose from. Similarly, most stenting techniques have improved, and interventional cardiologists have gained experience in routine stenting.

Our systematic review has several limitations. First, our results were based on aggregate participant data and thus we could not explore the effects of the different patient characteristics and stenting techniques based on outcomes. Second, among the 602 studies that compared stenting strategies, only nine articles were eligible for inclusion based on our definition of bifurcation lesion. Of the studies excluded, many did not clearly define bifurcation lesion size in the SB or included patients with an affected SB of a smaller diameter. Third, despite the vast amount of published studies, we found only a few trials conducted on patient with bifurcation lesions i.e. SB diameter of ≥ 2.25 mm for our review. Finally, since the TSB was recently approved by the FDA when this review was conducted, there was only one published prospective clinical trial that used the TSB.

## Conclusions

Our study did not find a difference between three different stenting strategies for bifurcation lesions. Additional clinical trials are needed comparing the three stenting strategies. These trials should include detailed data on the anatomy and severity of the bifurcation lesion as well as the type of stents deployed.

## Supplementary Information



## Figures and Tables

**Table 1:** Included Studies and Baseline Characteristics

Reference	Treatment Arms	Sample Size, n	Age, yrs	Male, %	Comorbidities, %				Follow-up, Months	Primary Endpoint
					Hypertension	MI	Diabetes	Smokers		
**Chen et al., 2011**	Provisional	185	64.6 ± 9.9	78.9	60.5	14.1	23.8	23.8	6, 12	MACE
	Complex	185	63.9 ± 11.1	76.2	65.4	17.3	19.5	30.8		
**Chen et al., 2014**	Provisional	2,552	65.0 ± 10.0	74.9	72.5	9.3	34.3	12.5	12	MACE
	Complex	1,108	68.0 ± 9.0	78.0	74.0	12.2	45.0	6.8		
**Chen et al., 2017**	Provisional	183	64.7 ± 10.0	75.8	60.9	14.2	23.1	NR	60	MACE
	Complex	183	63.9 ± 11.1	78.8	65.2	17.4	19.6	NR		
**Colombo et al., 2009**	Provisional	173	67.0 ± 10.0	76.3	79.8	35.3	22.0	16.8	6	MACE
	Complex	177	65.0 ± 10.0	80.2	70.6	44.6	23.7	20.3		
**Ferenc et al., 2008**	Provisional	101	66.7 ± 9.2	79.4	92.1	18.8	25.7	9.9	9	MACE
	Complex	101	66.9 ± 10.5	78.2	89.1	20.8	18.8	13.9		
**Généreux et al., 2015**	Provisional	349	64.6 ± 9.4	73.4	73.6	37.8	30.0	15.2	9	TVF
	Tryton	355	64.5 ± 10.6	71.8	73.2	28.1	23.9	17.5		
**Généreux et al., 2016**	Provisional	143	65.2 ± 9.2	81.8	76.8	40.4	28.7	15.5	9	TVF
	Tryton	146	64.5 ± 10.7	79.5	68.5	29.7	25.3	17.1		
**Hildick-Smith et al., 2010**	Provisional	250	64.0 ± 10.0	77.0	57.0	23.0	13.0	17.0	9	Composite Index*
	Complex	250	64.0 ± 11.0	77.0	62.0	25.0	11.0	17.0		
**Hildick-Smith et al., 2016**	Provisional	103	62.9 ± 10.8	85.0	63.0	39.0	25.0	56.0	12	Composite Index*
	Complex	97	63.5 ± 12.1	78.0	68.0	41.0	31.0	50.0		

Values are n (%) or Mean ± SD; NR: Not Reported; MI: Myocardial Infarction; MACE: Major Adverse Cardiac Event; TVF: Target Vessel Failure; Composite Index: all-cause death, MI, and TVF; Composite Index *: all-cause death, MI and target vessel revascularization

**Table 2:** Stenting Strategy Descriptions

Reference	Treatment Arms	Strategy Definition	Stent Used	Eluted Drug	Platform
**Chen et al., 2011**	Provisional	Provisional	EXCEL	Sirolimus	Biodegradable polymer
	Complex	DK CRUSH			
**Chen et al., 2014**	Provisional	Provisional	CYPHER, FIREBIRD, FIREBIRD-2, EXCEL, BIOMATRIX FLEX, PARTNER, XIENCE and ENDEAVOR	Sirolimus, Paclitaxel, Everolimus, Zotarolimus	Biodegradable polylactic-acid polymer, stainless steel, cobalt chromium
	Complex	Left to physician’s discretion			
**Chen et al., 2017**	Provisional	Provisional	EXCEL	Sirolimus	Biodegradable polymer
	Complex	DK CRUSH			
**Colombo et al., 2009**	Provisional	Provisional	CYPHER	Sirolimus	Stainless steel
	Complex	CRUSH			
**Ferenc et al., 2008**	Provisional	Provisional	CYPHER	Sirolimus	Stainless steel
	Complex	Routine T-stenting			
**Généreux et al., 2015**	Provisional	Provisional	DES commercially available in the US	Not specified	Cobalt chromium
	Tryton	Tryton	TRYTON + DES commercially available in the US		
**Généreux et al., 2016**	Provisional	Provisional	DES commercially available in the US	Not specified	Cobalt chromium
	Tryton	Tryton	TRYTON + DES commercially available in the US		
**Hildick-Smith et al., 2010**	Provisional	Provisional	TAXUS	Paclitaxel	Stainless steel
	Complex	Crush or Culotte; Left to physician’s discretion			
**Hildick-Smith et al., 2016**	Provisional	Provisional	NOBORI	Biolimus	Biodegradable polymer
	Complex	Culotte			

DES: Drug Eluting Stent; DK: Double Kissing
