# Supplementary material for: A Systematic Literature Review of Three Stenting Strategies for Bifurcation Lesions in Coronary Artery Disease
Source: J Health Econ Outcomes Res. 2019 Apr 26;6(2):95–105. doi: 10.36469/9746 (PMC7299450; doi:10.36469/9746)
Supplement: Supplementary file 1 [file jheor-6-2-9746-s01.pdf]

JHEOR Journal of Health Economics and Outcomes Research

OPEN ACCESS

Supplementary Online Content

Parsley-Raines L, Brandt DM, Carr DL, *et al*. A systematic literature review of three stenting strategies for bifurcation lesions in coronary artery disease. *JHEOR*. 2019;6(2):95-105.

Appendix A. Search Strategy
Appendix B. Data Extraction Form
Appendix C. Citations of Included Articles

This supplementary material has been provided by the authors to give readers additional information about their work.

Appendix A. Search Strategy

Medline	
Search Terms	coronary AND stent AND bifurcat* AND trial AND (compar* OR versus)
Publication Date	January 2007- July 2017
Filters Activated	Abstract available
Language	English

Cochrane	
Search Terms	coronary AND stent AND bifurcat* AND (compar* OR versus)
Publication Date	2007-2017
Filters Activated	Trials

Web of Science	
Search Terms	coronary AND stent AND bifurcat* AND trial AND (compar* OR versus)
Publication Date	2007-2017
Language	English
Document Type	Article
Indexes	All
Database	Web of Science Core Collection

Embase	
Search Terms	coronary AND stent AND bifurcat* AND (compar* OR versus)
Publication Date	2007-2017
Language	English only
Document Type	Article, Article in press
Indexes	All
Filters Activated	Controlled clinical trial, Randomized controlled trial

Supplemental Appendix

Appendix B. Data Extraction Form

**REF ID:**	**REF ID listed*
**General Information**	
Title:	**copy and paste title HERE**
Report reference:	**copy and paste reference HERE**
Publication type:	*i.e. Full article, Abstract, Conference Proceeding*<br>**If not 'Full article', STOP and exclude for Level 1*
Publication year:	
Geographic location:	**country of study, if known**
Interventional/Observational?	*Was this study observervational or was there an internvention, i.e. treatment, medication, procedure, etc.? *If 'Observational', STOP and exlcude from Level 1*
**Eligibility**	
Provisional stenting strategy? (Y/N)	*Does this article include a provisional stenting strategy?*<br>**If 'N', STOP and exclude from Level 1*
Comparator arm? (Y/N)	*Does this article compare the provisional stenting strategy to a NON-PROVISIONAL (i.e. Complex, Tryton) stenting strategy? *If 'N', STOP and exclude from LEVEl 1*
Prospective study? (Y/N)	*Is this study a prospective or restrospective?*<br>**If 'N' i.e. retrospective or other, STOP and exclude from Level 1*
6+ month follow up (Y/N)	*Does this study have a follow-up period that is 6 months or greater? *If 'N', STOP and exclude from Level 1*
Human subjects? (Y/N)	**If 'N', STOP and exclude from Level 1*
Bifurcated lesions? (Y/N)	*Does this article mention 'bifurcated lesions'? *If 'N', STOP and exclude from Level 1*
Decision: Include or Exclude?	*If you answered 'N' to any of the Eligibility criteria, Exclude. If you answered 'Y' to ALL, Include.*
Reason for exclusion?	*Put reasoning for exclusion i.e. abstract, no comparator arm, etc.*
Notes	*Add any additional notes about article or screening process HERE*
**** DO NOT PROCEED IF STUDY EXCLUDED FROM REVIEW ****	
**Selection Bias**	
Are the individuals selected to participate in the study likely to be representative of the target population?<br>What percentage of selected individuals agreed to participate?	
**Study Design**	
Type of study design:<br>Was the study described as randomized?<br>If Yes, was the randomization method described?<br>If Yes, was the method appropriate?	
**Confounders**	
Were there important differences between groups prior to the intervention?<br>If Yes, indicate the percentage of relevant confounders that were controlled (either in the design (e.g. stratification, matching) or analysis)?	
**Blinding**	
Was (were) the outcome assessor(s) aware of the intervention or exposure status of participants?<br>Were the study participants aware of the research question?	
**Data Collection Methods**	
Were data collection tools shown to be valid?<br>Were data collection tools shown to be reliable?	
**Withdrawals and Dropouts**	
Were withdrawals and drop-outs reported in terms of numbers and/or reasons per group?<br>Indicate the percentage of participants completing the study.	

Appendix B. Data Extraction Form - cont.

**Intervention Integrity**				
What percentage of participants received the allocated intervention or exposure of interest?<br>Was the consistency of the intervention measured?<br>Is it likely that subjects received an unintended intervention (contamination or co-intervention) that may influence the results?				
**Analyses**				
Indicate the unit of allocation<br>Indicate the unit of analysis<br>Are the statistical methods appropriate for the study design?<br>If the analysis performed by intervention allocation status (i.e. intention to treat) rather than the actual intervention received?				
**Methods**				
Objective	*Copy and Paste objective (aim of study)*			
Type of study (e.g. RCT, Cohort, Case-Control)				
Randomized?	*Were subjects randomized?*			
# sites (if applicable)				
**Risks**				
Representative sample?	*Yes/No*			
Participation Agreement	%			
Confounders mentioned?	Were controlled for	*Report the confounders mentioned in the article that they controlled for via stratification, matching, etc.*		
	Weren't controlled for	*Report the confounders mentioned in the article that they did not control for (probably mentioned in limitations paragraph)*		
	Blinding (e.g. single, double, none)			
**Define Group 1**	*How they defined their groups (e.g. DES, Tryton side-branch stent, BMS)*			
**Define Group 2**	*How they defined their groups (e.g. DES, Tryton side-branch stent, BMS)*			
**Define Group 3**	*How they defined their groups (e.g. DES, Tryton side-branch stent, BMS)*			
**Participants**		**Group 1**	**Group 2**	**Group 3**
# participants	n			
	%			
Age	Mean			
	Min			
	Max			
Gender				
Male	n			
	%			
Female	n			
	%			
Comorbidities				
Hypertension	n			
	%			
Peripheral Vascular Disease	n			
	%			
Myocardial infarction	n			
	%			

Appendix B. Data Extraction Form - cont.

**Participants**		**Group 1**	**Group 2**	**Group 3**
Comorbidities				
Diabetes	n			
	%			
Chronic Pulmonary Disease	n			
	%			
Renal insufficiency	n			
	%			
**Outcomes**				
Follow-up period	Mean			
	Min			
	Max			
Procedural time	Mean			
Serious AE				
Cardiac death	n			
	%			
Stent thrombosis	n			
	%			
Early myocardial infarction	n			
	%			
Myocardial infarction	n			
	%			
Drug-Eluting Stents	n			
	%			
Bare-Metal Stents	n			
	%			
Target Vessel Failure	n			
	%			
Target Lesion Revascularization	n			
	%			
Target Vessel Revascularization	n			
	%			
Procedural success	n			
	%			
MACE	n			
	%			

Appendix C. Citations of Included Articles

[1] Chen S-L, Santoso T, Zhang J-J, Ye F, Xu Y-W, Fu Q, *et al*. A randomized clinical study comparing double kissing crush with provisional stenting for treatment of coronary bifurcation lesions. *J Am Coll Cardiol*. 2011;57(8):914-20.

[2] Chen SL, Santoso T, Zhang JJ, Ye F, Xu YW, Fu Q, *et al*. Clinical Outcome of Double Kissing Crush Versus Provisional Stenting of Coronary Artery Bifurcation Lesions. *Circ Cardiovasc Interv*. 2017;10(2).

[3] Chen SL, Sheiban I, Xu B, Jepson N, Paiboon C, Zhang JJ, *et al*. Impact of the Complexity of Bifurcation Lesions Treated With Drug-Eluting Stents The DEFINITION Study (Definitions and impact of complEx biFurcation lesIons on clinical outcomes after percutaNeous coronary IntervenTIOn using drug-eluting steNts). *JACC Cardiovasc Interv*. 2014;7(11):1266-76.

[4] Colombo A, Bramucci E, Saccà S, Violini R, Lettieri C, Zanini R, *et al*. Randomized study of the crush technique versus provisional side-branch stenting in true coronary bifurcations: The CACTUS (Coronary bifurcations: Application of the Crushing Technique Using Sirolimus-eluting stents) study. *Circulation*. 2009;119(1):71-8.

[5] Ferenc M, Gick M, Kienzle R, Bestehorn H, Werner K, Comberg T, *et al*. Randomized trial on routine vs. provisional T-stenting in the treatment of de novo coronary bifurcation lesions. *Eur Heart J*. 2008;29(23):2859-67.

[6] Généreux P, Kini A, Lesiak M, Kumsars I, Fontos G, Slagboom T, *et al*. Outcomes of a dedicated stent in coronary bifurcations with large side branches: A subanalysis of the randomized TRYTON bifurcation study. *Catheter Cardiovasc Interv*. 2016;87(7):1231-41.

[7] Généreux P, Kumsars I, Lesiak M, Kini A, Fontos G, Slagboom T, *et al*. A Randomized Trial of a Dedicated Bifurcation Stent Versus Provisional Stenting in the Treatment of Coronary Bifurcation Lesions. *J Am Coll Cardiol*. 2015;65(6):533-43.

[8] Hildick-Smith D, Behan MW, Lassen JF, Chieffo A, Lefevre T, Stankovic G, *et al*. The EBC TWO Study (European Bifurcation Coronary TWO): A Randomized Comparison of Provisional T-Stenting Versus a Systematic 2 Stent Culotte Strategy in Large Caliber True Bifurcations. *Circ Cardiovasc Interv*. 2016;9(9):8.

[9] Hildick-Smith D, de Belder AJ, Cooter N, Curzen NP, Clayton TC, Oldroyd KG, *et al*. Randomized Trial of Simple Versus Complex Drug-Eluting Stenting for Bifurcation Lesions The British Bifurcation Coronary Study: Old, New, and Evolving Strategies. *Circulation*. 2010;121(10):1235-43.
